# Preferences of Patients With Musculoskeletal Disorders Regarding the Timing and Channel of eHealth and Factors Influencing Its Use: Mixed Methods Study

**DOI:** 10.2196/44885

**Published:** 2023-09-27

**Authors:** Jeffrey van der Ven, Bart J F van den Bemt, Liset van Dijk, Merel Opdam, Lex L Haegens, Johanna E Vriezekolk, Lise M Verhoef

**Affiliations:** 1 Department of Research Sint Maartenskliniek Ubbergen Netherlands; 2 Radboud University Medical Centre Nijmegen Netherlands; 3 Nivel, Netherlands Institute for Health Services Research Utrecht Netherlands

**Keywords:** eHealth, telehealth, telemedicine, chronic diseases, chronic illness, musculoskeletal disorders, multiple methods, perspectives, preferences, citizen science, digital hospital services, musculoskeletal, orthopedic, citizen, civic, society, health tech, Capability, Opportunity, Motivation and Behavior Model, COM-B, focus group, rheumatoid arthritis, arthritis, rehabilitation, kinesio, physio, rheuma, thematic analysis, semistructured interview

## Abstract

**Background:**

Implementation of eHealth is progressing slowly. In-depth insight into patients’ preferences and needs regarding eHealth might improve its use.

**Objective:**

This study aimed to describe when patients want to use eHealth, how patients want to communicate and receive information digitally, and what factors influence the use of eHealth in clinical practice.

**Methods:**

A multimethod study was conducted. Two meetings of ~5.5 hours with plenary information sessions and focus groups were held with 22 patients from the rheumatology, orthopedics, and rehabilitation departments of a Dutch hospital specialized in musculoskeletal disorders. Assignments were performed during the focus groups in which qualitative (eg, semistructured interview questions) and quantitative (ie, voting and ranking factors) data were collected.

**Results:**

The way patients want to use eHealth varies between patients and moments of a patient’s care pathway. Patients’ digital channel preferences depended on the need for interaction with a health care provider (HCP). The interaction need is in turn influenced by the degree to which information or communication is specific to an individual patient and leads to consequences for the patient. The 5 most important factors influencing the use of eHealth were access to medical information (eg, electronic health records), perceived control over disease management, correctness and completeness of information, data security, and access to information or an HCP at any time. The 5 least important factors influencing eHealth use were help with using digital devices, having internet or equipment, digital skills, attitude or emotions toward eHealth, and societal benefits.

**Conclusions:**

Patients identified opportunities for using eHealth during all moments of their care pathway. However, preferences for eHealth varied between patients and phases in the care pathway. As a consequence, eHealth should be tailored to fit individual patients’ preferences but also the need for interaction regarding different topics by offering a variety of digital channels with a gradient of interaction possibilities. Furthermore, digital skills and access to the internet might become less important to focus on in the future. Improving eHealth use by patients may be achieved by providing patients access to correct and safe (medical) information and more control over their care.

## Introduction

In the past 2 decades, and especially recently during the COVID-19 pandemic, it has become apparent that the use of digital information and communication technologies in health care (eHealth) has the potential to provide great benefits [1,2]. eHealth, defined as the application of both digital information and communication to support and improve health and health care [3], can make health care more independent of staff, time, and location. This might foster the efficiency and patient-centeredness of health care, for instance, through intensification of home monitoring and tailoring information to personal needs [4-6]. Furthermore, the deployment of eHealth can lead to more efficient delivery of care and therefore contribute to an affordable and sustainable health care system [4,7]. This is needed as health care costs are rising due to the increasing availability of novel (expensive) treatment options, the aging of the population, and the subsequent increase of costs for treatment of chronic diseases and long-term (secondary) care [8,9]. Furthermore, a shortage of health care providers (HCPs) is expected in the future [10,11]. These developments indicate the need for a (digital) transformation of the health care system [12,13]. Although eHealth should be a means and not an end in itself, it can be an important tool to keep health care affordable and accessible, and strengthen the position of patients with chronic diseases in secondary care [4,7,14,15].

Similar to eHealth applications in general, the use of eHealth in secondary care settings is advancing slowly [16-18]. Important barriers to the implementation of eHealth are insufficient funding and concerns about privacy [1,19]. Furthermore, the lack of patient involvement in innovations is seen as a barrier [20,21]. Studies indicate that technologies are more likely to be successful when they meet patients’ needs and are based on factors that influence the eHealth use of patients [18,22]. Therefore, patient involvement might become an important impulse for the broad-scale implementation of eHealth by gaining insight into patient-level factors influencing its implementation for example [17,20].

However, it is unclear when patients with chronic conditions want to use eHealth and what digital channels (eg, website, email, video call) they prefer during different moments in their care pathway [23]. Insight into patient factors (and their importance) that influence the use of eHealth can inform hospitals on future directions regarding the implementation of eHealth and patient-centered care.

Therefore, we aimed to answer the following research questions (RQs): (1) WHEN do patients think eHealth is suitable during various phases of their care pathway? (2) HOW do patients want to communicate or receive information digitally? (3a) WHAT are factors influencing the use of eHealth? and (3b) WHAT is the relative importance of these factors influencing the use of eHealth according to patients?

To answer these research questions, we studied patients with musculoskeletal disorders (MSDs) in a hospital specialized in treating MSDs, which is a category of diseases with a high rising burden on the health care system [9,24]. Participants recruited from a large group of patients with a variety of chronic conditions and associated high costs might serve as a model for other populations with chronic diseases.

## Methods

### Study Design and Setting

**Figure 1:**
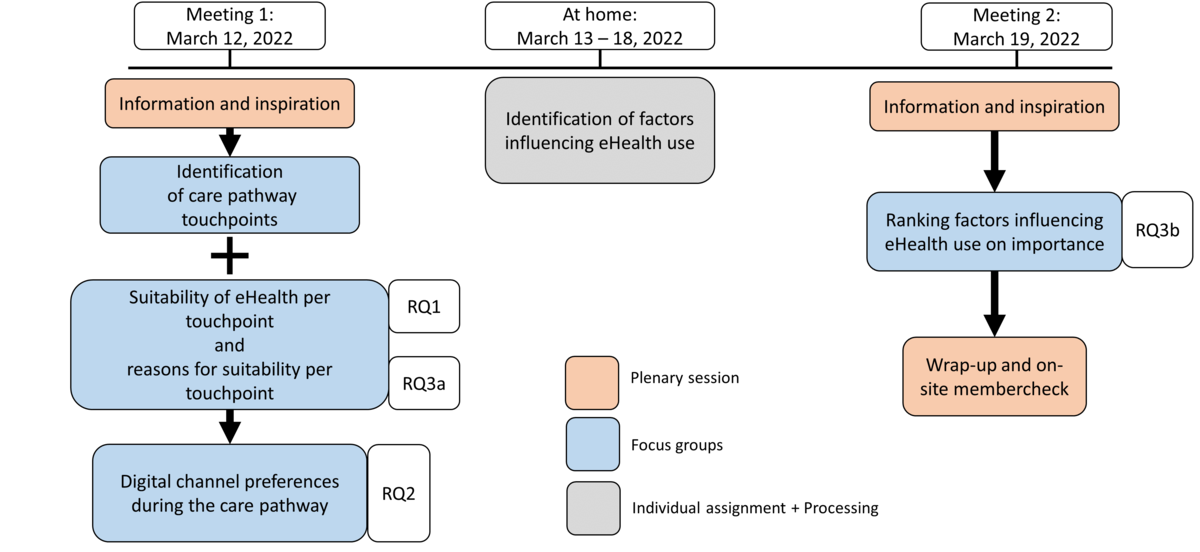
Overview of data collection. The data collection steps that were used during this study. Arrows indicate the order of the steps performed. The legend indicates the type of session and data collection. RQ: research question.

To answer our research questions, a multimethod study design was most suitable [25]. Specifically, we chose to use the “citizen platform method” in which citizens (in our case patients) are inspired and informed about a complex subject and are subsequently asked to share their experiences, opinions, and preferences. This method was originally developed by NICE and adapted to the Dutch setting by Nivel [26,27]. This method was deemed appropriate because the use and implementation of eHealth are complex issues and, therefore, require properly informed participants and multiple days of research to gain in-depth insights. The study was conducted at the Sint Maartenskliniek in Nijmegen, the Netherlands, which is a Dutch hospital specializing in MSDs. Two subsequent meetings (of ~5.5 hours) with the same participants were organized in March 2022, 1 week apart from each other, with a short homework assignment in between (Figure 1).

For both days, a different expert in digital (health) technology was invited to inform and inspire participants about eHealth during plenary sessions. Focus groups with assignments were designed by the study team according to this study’s aims. To this end, the Capability, Opportunity, Motivation, and Behavior Model (COM-B) [28] was used to systematically identify factors influencing behavior, that is, the use of eHealth, as was done in previous studies investigating eHealth use [29,30]. The program overview can be found in Figure 1 and the timetable in Multimedia Appendix 1. The plenary sessions were moderated by a researcher with expertise in qualitative focus group methods (BJFvdB). Six other researchers with moderate (JvdV, LLH, and MO) to advanced experience (LMV, JEV, and LvD) in qualitative research were present to facilitate 4 parallel focus groups with assignments. No prior relationship with the participants was established before the start of the study.

Two patients with rheumatoid arthritis were involved as patient research partners. The patient research partners advised the study team during participant recruitment and the development of the assignments.

### Participant Recruitment

Ambulatory patients with a therapeutic relationship with an HCP from the Sint Maartenskliniek in Nijmegen were recruited from the departments of rheumatology, orthopedics, and rehabilitation. Recruitment was conducted through (1) a user panel of patients who provide feedback on the development of the hospital's patient portal and (2) through HCPs of the departments of rheumatology, orthopedics, and rehabilitation. Patients were eligible when they were 18 years or older, had sufficient understanding of the Dutch language, had an MSD and initiated treatment for that condition in the hospital, had a therapeutic relationship with an HCP from the Sint Maartenskliniek in Nijmegen, and were able and willing to sign an informed consent. Purposive sampling based on age, sex, diagnosis, disease duration, and digital skills was used to increase the chance to gather a broad range of opinions and views on the topic. In total, a maximum of 25 participants was aimed for, as this was advised as a suitable number of participants by an expert in the Citizen Platform method (LvD) [26,27]. Participants were reimbursed for their travel expenses and additionally received a €50 (US $55.26) gift card for their time and effort.

### Data Collection and Analysis

#### Overview

Participants’ characteristics (age, sex, duration of disease, and diagnosis) were collected from the electronic health records. Marital status, education, employment status, travel distance to the clinic, health literacy (using the health literacy short form-12 [31]), a brief inventory of digital skills [32], and prior experience with the hospital’s patient portal and video consultations were collected through a short web-based or postal survey, depending on the participants’ preference.

Data regarding the research questions were collected during focus groups with assignments (Figure 1). In between focus groups, researchers collated and summarized the findings from each assignment. These aggregated results were used as input for the next assignment and to standardize questioning in each focus group. A topic list for qualitative assignments is provided in Multimedia Appendix 2. The data collection, results, and discussion are structured into four recurring paragraphs related to the research questions:

- RQ1: WHEN—Patient preferences for communication method during different phases in a care pathway.
- RQ2: HOW—Patient preferences for digital communication channels during a care pathway.
- RQ3a: WHAT—Factors influencing the use of eHealth during the various phases of a care pathway.
- RQ3b: WHAT—Relative importance of factors influencing the use of eHealth.

#### RQ1: WHEN

First, a care pathway map of touchpoints, defined as every interaction between patient and health care either passive (eg, uploading info into the patient portal) or active (eg, consultation with an HCP), was created. Subsequently, quantitative data related to when patients want to use eHealth were collected by participants voting for their preferred communication method (ie, *digital*=exclusively through eHealth, *F2F*=face-to-face communication but also including written paper information, or *hybrid*=a combination of the definitions of the previous explanations) for each touchpoint. Results were summarized and displayed.

#### RQ2: HOW

Qualitative data related to how patients want to communicate digitally were collected by inviting participants to mention any digital channel of their liking for each touchpoint and explore reasons for a preference. Audio recordings were summarized by 1 author, and the summary was verified by another author. Finally, a consensus-based summary was drafted after a discussion between 3 of the authors. The final summary was verified in the audio recordings by the author who initially summarized the recordings. Results were descriptively reported, but not transcribed and coded, as the answers given by patients were not extensive enough for a full thematic analysis.

#### RQ3a: WHAT

Qualitative data related to what factors influence eHealth use by patients were collected by semistructured questions exploring why participants chose a communication method for a certain touchpoint. Audio recordings were transcribed verbatim and inductively coded in ATLAS.ti (version 9.1.6; ATLAS.ti Scientific Software Development GmbH) by 2 researchers independently according to the 6 phases approach advised by Braun and Clarke [33] (Multimedia Appendix 3).

#### RQ3b: WHAT

Factors influencing the use of eHealth use and their importance were collected with a mixed methods approach. First, we gathered factors stimulating or hindering the use of eHealth with a short questionnaire (Multimedia Appendix 4). Results were thematically categorized by 2 researchers (JvdV and LMV) into factors. Participants individually ranked these factors (printed on cards) according to the Q-methodology (Figure S1 in Multimedia Appendix 5). Results were summarized and displayed as the mean (SD) and range of points given per factor by all participants.

After all focus group assignments, the plenary moderator (BJFvdB) summarized the findings from the assignments during a plenary session and participants were invited to provide feedback. Subsequently, participants were thanked for their participation, and gift cards and reimbursements were handed out. Anonymous evaluation forms were filled in to evaluate the meetings, including an overall satisfaction scale from 1 to 10 (1 being very unsatisfied and 10 very satisfied). The results of each assignment were checked by all focus group moderators afterward to ensure consistency in analysis and interpretation.

### Ethical Considerations

Ethical approval was waived by the Medical Research Ethics Committee of Eastern Netherlands as this study did not meet the criteria for the Medical Research Involving Human Subjects Act (file: 2021-13283). All participants gave written informed consent for their participation. Transcribed data were anonymized and coded so that the analysis did not contain identifiable patient information. Data were handled according to the Dutch General Data Protection Regulation.

## Results

### Overview

A total of 22 participants participated in the study (Table 1). One participant was present only during the first day and another participant only during the second day; therefore, 21 participants participated during each day. Participants from the rheumatology outpatient department included patients with inflammatory rheumatic diseases, osteoarthritis, and osteoporosis. While the majority of them were receiving pharmacological treatment at the time of the study, some were under observation on an outpatient basis without active treatment. Participants from the orthopedics department included patients who had undergone surgery for osteoarthritis or other joint abnormalities, mainly in the lower extremities. Participants from the rehabilitation department included patients with a neuromuscular disorder and 1 amputee (Table 1). All participants indicated on the evaluation form that the content of both days was understandable to them. The average score given for the overall days was an 8 out of 10 (range 7-9).

**Table 1:** Characteristics of the study population (N=22).

Characteristics			Values
Sex (male), n (%)			13 (59)
Age (years), mean (SD)			67.4 (10.6)
**Marital status, n (%)**			
	Married	14 (64)	
	Divorced	2 (9)	
	Never married	2 (9)	
	Widower	4 (18)	
**Level of education,^a^ n (%)**			
	Low	4 (18)	
	Medium	6 (27)	
	High	12 (55)	
**Employment status, n (%)**			
	Employed	4 (18)	
	Retired	10 (46)	
	Fulltime housewife/husband	2 (9)	
	Unfit for work	6 (27)	
**Distance to the hospital (km), n (%)**			
	0-25	12 (55)	
	25-50	2 (9)	
	50-75	4 (18)	
	>75	4 (18)	
**Diseases,^b^ n (%)**			
	Inflammatory rheumatic disorders (eg, rheumatoid arthritis and psoriatic arthritis)	17 (77)	
	Osteoarthritis	10 (45)	
	Skeletal disorders and joint abnormalities (eg, hallux valgus and scoliosis)	8 (36)	
	Neuromuscular disorders (eg, postpolio syndrome and spinal cord injury)	5 (23)	
	Amputee	1 (5)	
	Osteoporosis	1 (5)	
Disease duration (years), median (IQR)			9 (4-13)
Health literacy SF12 index,^c^ mean (SD)			32.7 (6.6)
**Owns a laptop, smartphone, or tablet, n (%)**			
	Yes	21 (95)	
	No	1 (5)	
**Searches health information on the internet, n (%)**			
	Yes	17 (77)	
	No	5 (23)	
**Uses email, n (%)**			
	Yes	19 (86)	
	No	2 (9)	
	With help from others	1 (5)	
**Uses apps, n (%)**			
	Yes	18 (82)	
	No	4 (18)	
**Downloads apps, n (%)**			
	Yes	19 (86)	
	No	2 (9)	
	With help from others	1 (5)	
**Experience with the patient portal, n (%)**			
	No	2 (9)	
	Little	2 (9)	
	Average	10 (46)	
	Much	8 (36)	
**Experience with video consultations, n (%)**			
	No	11 (50)	
	Little	4 (18)	
	Average	4 (18)	
	Much	2 (9)	
	A lot	1 (5)	

^a^Level of education: Low—up to and including lower vocational training; medium—up to and including secondary vocational training; higher—including higher vocational training and university.

^b^Diseases: some participants have multiple conditions; therefore, the total exceeds 22, and and percentages do not add to 100.

^c^The health literacy index ranges from 0 to 50, the latter being the highest possible value (ie, having the highest health literacy).

### RQ1: WHEN—Patient Preferences for Communication Method During Different Phases in a Care Pathway

**Figure 2:**
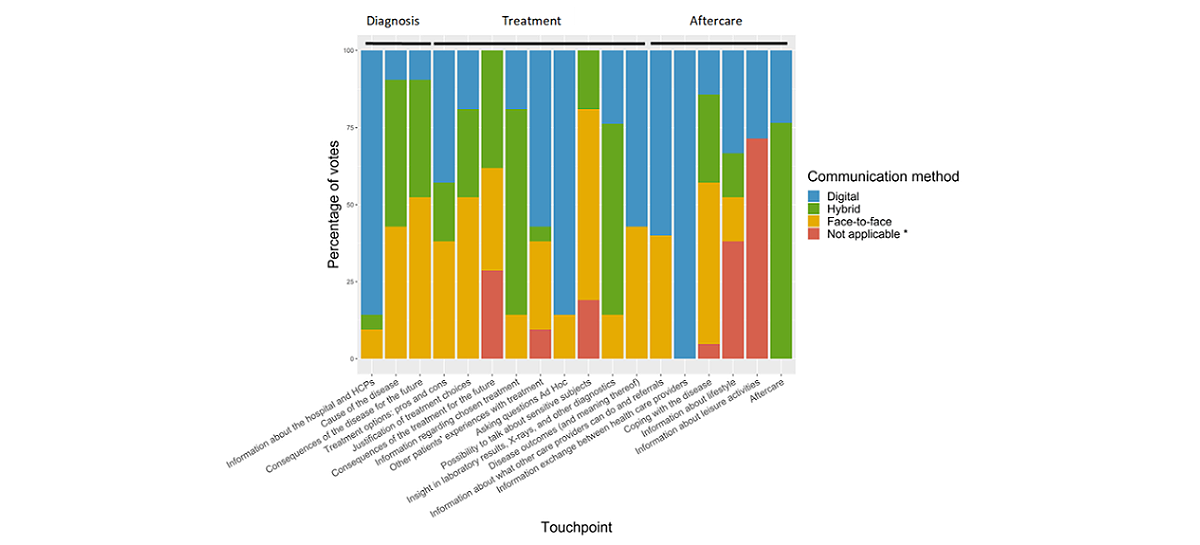
Preferences for communication method per touchpoint. Touchpoints during a care pathway that were identified by participants. The communication method shows patients’ preferences for a digital, hybrid, or face-to-face way of receiving information or interacting. *This touchpoint was perceived as irrelevant by a group of participants. HCP: health care provider.

During the first assignment, 18 touchpoints in a possible care pathway were identified (presented on the x-axis of Figure 2). In the second assignment, participants voted for their preferred method of communication (digital, hybrid, or F2F) for each touchpoint, and the results are depicted in Figure 2. Preferences for communication methods differed between participants but also between touchpoints. For each touchpoint in a care pathway, there were possibilities for using eHealth. For the consequences of the treatment for the future and the possibility of talking about sensitive subjects, only F2F or hybrid was voted for.

### RQ2: HOW—Patient Preferences for Digital Communication Channels During a Care Pathway

**Figure 3:**
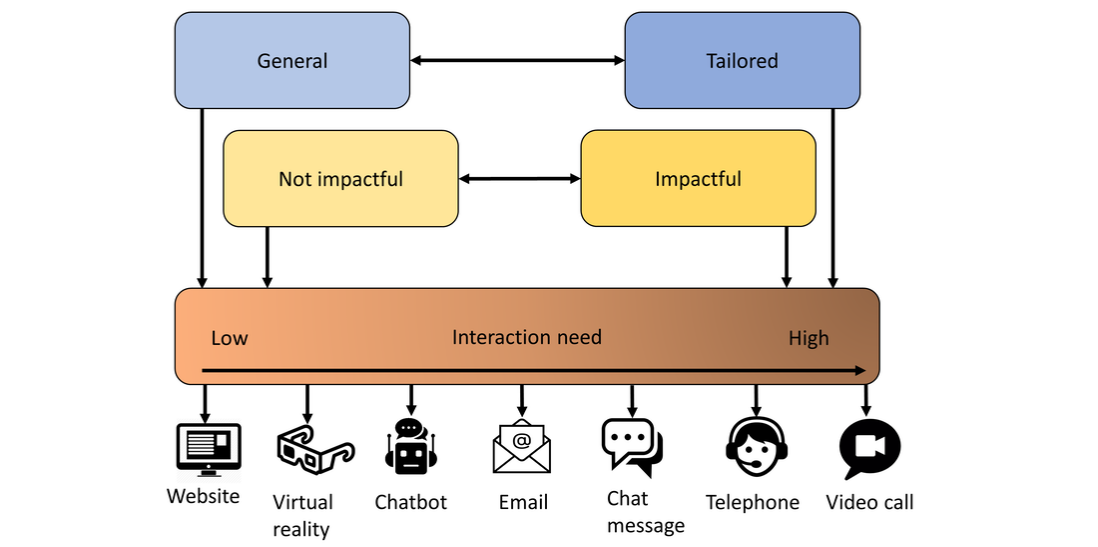
Types of information and influence on digital channel preference. The types of information (general, tailored, impactful, and not impactful) and the interaction need (low and high interaction need on the left and right, respectively). General and not impactful information influences a low interaction need and tailored and impactful information a high interaction need, which are indicated by arrows.

Participants reported that their preferred digital channel depended on the interaction need (Figure 3). Figure 3 shows the perceived interactions between digital channel, interaction need, and characteristics of information and communication.

Tailored information or topics were in our study defined as information that is specific to an individual patient, such as disease outcomes and meaning thereof specific to an individual. For tailored information, the interaction need was higher compared to general topics (eg, information about hospitals and HCPs), and therefore participants preferred to converse with an HCP often through video calling as a preferred digital channel. However, tailored or personal information should also be available in the hospital’s digital patient portal according to participants. The interaction need was lower for general topics compared to tailored topics and participants preferred to look up or read general information on the hospital website. Furthermore, participants indicated that they wanted to make use of chat messages, a chatbot, or email for asking general or practical questions, supporting the lower need for interaction for general information. Finally, in 1 group, the use of virtual reality was discussed as a way of discovering the cause of the disease in the human body.

Impactful information or topics were, in our study, defined as medical information leading to substantial consequences for the patient, such as the consequences of treatment for the future or discussing the treatment options touchpoint. For impactful information, the interaction need was higher than for not impactful topics (eg, information about lifestyle) and participants preferred to discuss these topics with an HCP with video calling as the most preferred digital channel. For information or topics that are not impactful, participants mentioned that chat messages, a chatbot, or a website (including the patient portal) would suffice. Reasons to choose email as a digital channel included aftercare messages with short questionnaires and receiving notifications for updates in the portal or time window in which an HCP will call for an appointment.

### RQ3a: WHAT—Factors Influencing the Use of eHealth During the Various Phases of a Care Pathway

#### Overview

Using thematic analysis, 8 themes were identified describing factors influencing eHealth use: (1) eHealth accessibility, (2) patient’s capability, (3) characteristics of eHealth, (4) perceived logistical benefits of eHealth, (5) empowerment, (6) characteristics of disease and treatment, (7) properties of the desired communication, and (8) properties of the information or message. Quotes supporting the themes can be found in Textbox 1.

**Quotes related to the thematic analysis of RQ3a.**

**eHealth accessibility**

- *Because I just work with my phone since I don’t have a computer. So I’d prefer to have it sent to me at home.*

**Patient’s capability**

- *That’s the problem: I have trouble with the computer.*
- *And many of them (patients) also find it difficult to work like that (with digital devices). People who are dyslectic also have a problem.*

**Characteristics of eHealth**

- *(When using eHealth) It’s your own surroundings and you can look at the information (about hospital and HCP) at your own pace. You can look at the photos (of the HCP) several times.*
- *Digitally. That way you can make the letters (of a prescription) more legible.*

**Perceived logistical benefits**

- *Then you can avoid some of that travelling back and forth but still combine being able to talk and look face-to-face without having to be present at the location. That’s possible nowadays, so we should definitely do it. That saves enormous amounts of bother and time for both parties.*

**Empowerment**

- *I very much liked knowing in advance what I could expect from the discussion. The discussion (about diagnosis) can become very serious if the results of the exam are D. It’s good to know in advance that the results (of a diagnosis) can be A, B, C or D, and I was very glad that I could prepare myself for this (digitally).*
- *Then (when reading a patient association’s forum) you find a lot of information (about consequences of the disease) that doesn’t apply to you yet. So I think it’s better to search for that information only when it becomes relevant to me.*

**Characteristics of disease and treatment**

- *You can do very simple exercises (physical therapy) digitally. I have a number of excercies lined up (on a mobile application) and I just play the list. (…) That’s digitally, but those are the simple exercises. If you have to be here for difficult physical therapy, it has to be here on location.*
- *That (receiving information about treament) completely depends on the patient in question and the sort of problem they have. One patient might say ‘I prefer to do that (receiving information about treatment) face-to-face with my physician in a separate room’ and another patient might say ‘an internet consultation is sufficient for me.*

**Properties of the desired communication**

- *You first have to build up a sense of trust (with the HCP). I don’t see my son every day, but it’s fine when I phone him. It’s all about knowing who the other person is.*
- *When you read the information (about the consequences of treatment) digitally, you understand it but it seems abstract; if you hear it in a consultation it makes more of an impact. Then it’s suddenly part of yourself.*

**Properties of the information and message**

- *If it’s about my specific treatment, like what are you going to remove from my bones, then I really want to ask the doctor that personally. But if it’s only about the intake procedure, what the expected recovery period is and other general information, I can search for that on the internet.*
- *I’ve already read a number of things (about consequences of treatment) online, but now I have a specific question about myself. So a lot of those things are hybrid. For example, I can find out online that I won’t be able to participate in a marching event anymore. But it’s the specific things that are difficult to find (digitally).*

#### eHealth Accessibility

eHealth accessibility consisted of several aspects. First, patients should have access to the right software and hardware to be able to use eHealth. Some participants did not own a certain device (like a smartphone) and some services are not yet available to every operating system. Furthermore, digital information needs to be visually or auditively accessible to patients. Finally, comprehensibility was perceived as a precondition for the accessibility of eHealth, enabling patients to fully understand the content of certain information.

#### Patient’s Capability

Patients have to be capable of using digital devices in order to use eHealth, both physically (eg, being capable of operating a digital device, and reading small letters) and mentally (eg, health literacy). Previous experience enhanced digital skills, as participants felt more capable due to using eHealth during the COVID-19 pandemic. Participants who were less digitally skilled sometimes received help from their children. Therefore, whether patients are capable of using eHealth can influence the actual use thereof.

#### Characteristics of eHealth

The possibility to have contact or access to information independent of place and time, for example, after an F2F appointment with an HCP positively influenced participants’ use of eHealth. This is because it allowed patients to process information at their own pace and in their own environment and review information as many times as necessary. eHealth also has positive and negative characteristics that can act as an influencing factor for using eHealth. Positive characteristics included digital data exchange between care providers, having digital information in one place, enlarging characters on digital devices (eg, prescription notes), and receiving notifications when new test results are uploaded in the patient portal. Negative characteristics included an overload of notifications and still having to be at home for video consulting, as opposed to a telephone call. In summary, specific characteristics of eHealth can influence patients’ preferences for using eHealth.

#### Perceived Logistical Benefits of eHealth

Logistical benefits of eHealth by patients included less traveling time, effort for both the patient and the HCP, and costs. Additionally, decreased use of paper was mentioned. Therefore, these benefits perceived by patients may act as a facilitator for choosing a digital or hybrid communication method.

#### Empowerment

Participants mentioned having more control over when, where, and if to access information regarding disease or treatment when using eHealth compared to conversations with HCPs. To illustrate, participants mentioned that they only wanted to receive information when it became applicable to them, such as certain side effects or experiences from other patients. Furthermore, participants expressed a need to prepare for a consultation by receiving digital information beforehand. Knowing what to expect during an F2F visit by preparing digital information could have a comforting effect as mentioned by many participants. Finally, eHealth can improve empowerment by facilitating shared decision-making regarding treatment options. Many participants appreciated reading about the treatment options digitally and subsequently making a treatment choice together with the HCP. To summarize, patients experience more control and empowerment over their care due to using eHealth, which acts as a facilitator for using eHealth.

#### Characteristics of Disease and Treatment

Participants indicated that physical therapy exercises can be done at home using a mobile app, after learning how to perform them with the physical therapist. Also, in the aftercare phase, participants indicated to be satisfied with digital communication under the condition to have F2F appointments at least once a year with their treating HCP. Hence, the severity of the specific condition, symptoms, and treatment contributed to the preference for a certain communication method.

#### Properties of Desired Communication

An important precondition in digital communication between the patient and the HCP is trust. Participants indicated that digital communication can be as good and personal as F2F, after getting to know an HCP. However, for some topics such as changing habitual behavior (eg, lifestyle changes) or making decisions regarding treatment, an F2F appointment might be necessary to obtain the impact that is needed. The possibility to discuss or ask questions also was important in the desired communication. Some participants preferred to ask questions F2F, but others preferred email or telephone. Finally, participants also expressed that digital data exchange between HCPs would be desirable. This way the patients do not need to supply the same information repeatedly to different HCPs. Therefore, the properties of the desired communication between patients and HCPs influenced preferences for a communication method.

#### Properties of Information or Message

For more severe (eg, consequences of the disease) or sensitive information (eg, sexuality), participants often preferred to converse F2F. In contrast, for information regarding general or less impactful subjects, digital sources were preferred. Participants mentioned that information relating to their personal situation was difficult to find digitally, and therefore preferred to receive this type of information from an HCP, often through F2F contact. Finally, information characteristics like up-to-dateness, completeness, reliability, security, and comprehensibility also influence patients’ willingness to use eHealth. In conclusion, the severity and sensitivity of information influenced the preference for digital or written information or F2F communication.

### RQ3b: WHAT—Relative Importance of Factors Influencing the Use of eHealth

Based on the homework assignment (assignment 4), 23 factors influencing the use of eHealth by participants were identified (Table 2). Participants ranked these 23 factors in terms of importance during the fifth assignment (Table 2). Access to medical information, perceived control over disease management, correctness or completeness of information, and data security were the highest-scoring factors influencing the use of eHealth. Attitude or emotions, digital skills, access to the internet or equipment, and receiving help were among the least important factors for patients in the use of eHealth.

**Table 2:** Relative importance of factors influencing the use of eHealth.^a^

Factor	Definition	Mean (SD)	Range
Access to medical information	The extent to which eHealth helps you to gain insight into and over medical information	1.59 (1.44)	0 to 4
Perceived control over disease management	The extent to which eHealth gives you more control over your health care (eg, making appointments by yourself)	1.22 (2.02)	−3 to 4
Correctness or completeness of information	The extent to which your medical/personal data are correct and complete	1.09 (1.34)	−1 to 4
Data security	The extent to which the storage and exchange of your (medical) information happens securely	1.05 (1.99)	−4 to 3
Access to information or an HCP^b^ at any time	The extent to which eHealth ensures you have access to information or an HCP at any time (eg, reading information at home or asking quick questions)	0.95 (2.15)	−3 to 4
Exchange of (medical) information between platforms/services/HCPs	The extent to which exchange of your (medical) information is possible, therefore not needing to give the same information twice (eg, between hospitals and HCPs)	0.91 (2.04)	−3 to 4
Keeping agreements	The extent to which agreements made with you are lived up to (eg, receiving an answer to a question within the specified time window)	0.86 (1.46)	−2 to 4
Usability	If the eHealth application is easy to use for you (eg, clear, appealing, ease of log-in methods)	0.68 (1.94)	−3 to 4
Comprehensibility of information	The extent to which information you receive through eHealth (eg, diagnostic test results) is comprehensible	0.59 (1.79)	−4 to 4
Feeling of personal contact	The extent to which contact through eHealth gives you a feeling of personal contact (eg, nonverbal communication)	0.55 (1.47)	−3 to 3
Accessibility of eHealth	The extent to which eHealth is accessible to use for you (eg, the preferred digital channel, availability on Android, IOS, and Windows)	0.32 (2.06)	−3 to 4
Functionalities of eHealth	What functionalities are available to you (eg, exercise portal, ordering medication, planning appointments, asking questions, and insight into test results)	0.26 (1.69)	−3 to 3
Working eHealth	If the eHealth applications work for you as they are supposed to	−0.10 (1.99)	−4 to 3
Timely usage of eHealth	If eHealth is or can be used at the right moments in your care pathway	−0.12 (1.76)	−3.33 to 4
Personal advantages	The extent to which eHealth provides benefits for you as a person (eg, saving time or travel costs, convenience)	−0.23 (2.02)	−3 to 4
Facultative	If you have a choice between digital channels and if the use of eHealth remains free of choice	−0.46 (1.97)	−4 to 4
Knowledge about eHealth	If you have adequate knowledge about eHealth to use it	−0.95 (1.99)	−4 to 4
Physically capable of using eHealth	If you are physically capable of using eHealth (eg, reading small text, operating a smartphone)	−1.23 (1.41)	−3 to 2
Societal benefits	The extent to which the use of eHealth provides a benefit for society (eg, reduction of CO_2_, reduction of health care costs)	−1.23 (1.54)	−4 to 2
Attitude or emotions	If your attitude and/or emotions towards eHealth influence your use thereof (eg, anxiety for using, trust, and positive experiences)	−1.33 (1.69)	−4 to 3
Digital skills	How well you can handle eHealth (eg, understanding how to use an application)	−1.40 (1.19)	−3.3 to 0
Access to internet or equipment	If you have an internet connection and are in the possession of a computer/smartphone/tablet	−1.59 (1.91)	−4 to 2
Help with using eHealth	The extent to which you receive help and a clear explanation to use eHealth	−2.05 (1.99)	−4 to 4

^a^23 factors influencing the use of eHealth including explanation and mean (SD) scores and range. The score is calculated as the mean of points given to each factor by 22 participants (Figure S1 in Multimedia Appendix 5). The range shows the minimal and maximal score that was given for a factor, respectively (range –4 to 4). Positive and negative scores indicate that the factor is considered important and unimportant to patients for eHealth use, respectively. A score around zero means that patients felt neutral about this factor for their use of eHealth.

^b^HCP: health care provider.

## Discussion

### Principal Findings

This paper provides insight into the patient’s perspective on eHealth using the innovative Citizen Platform method: patients with MSDs perceive opportunities for eHealth during each touchpoint of their care pathway. Furthermore, we show that there is large variability in preferences between patients and between moments in the care pathway for using eHealth and how tailored and impactful information influences digital channel preferences. Finally, we provide evidence on the factors that are involved in the use of eHealth and their relative importance.

For RQ1, WHEN, patients were almost never unanimous when voting for their preferred communication method throughout their care pathway, indicating considerable differences between patients with MSDs. This is consistent with other studies, where differences in the use of eHealth are explained by patient demographics, such as ethnicity, age, income, and education [34,35]. Furthermore, preferences also varied strongly between touchpoints, defined as every interaction between patient and health care either passive (eg, uploading info into the patient portal) or active (eg, consultation with an HCP). However, patients did see possibilities for using eHealth during all moments in their care pathway. As variation in the preferred communication method existed between patients and between moments in their care pathway, the communication method should be aligned with each patient’s needs and preferences individually.

For RQ2, HOW, overall, patients reported that their preferred digital channel depended on the need for interaction with an HCP. Therefore, telephone or video-based eHealth might be more suited when patients have higher needs for interaction with an HCP (eg, when discussing treatment options or the consequences of the disease). A website or chatbot might be more suitable when patients do not feel the need to talk directly with an HCP (eg, when reading information about the hospital or lifestyle). Furthermore, we show that these preferences for digital channels are based on the degree to which information is specific or impactful. Translating these results into practice implies that a wide variety of eHealth applications with a gradient of interaction possibilities should be offered in routine care for patients with chronic diseases and channels used should be guided by the degree of specificity and impact a message has.

For RQ3a and RQ3b, WHAT, several factors influencing the use of eHealth were observed including capability (eg, reading small letters and health literacy), accessibility (eg, owning a device, visual or auditive accessibility), and characteristics of eHealth itself and perceived benefits, which has been found in previous research [1,36]. Additionally, many patients were open-minded to receiving at least a part of their care in a hybrid or digital form, especially when a bond of trust was created with an HCP. The use of eHealth also depended on contextual factors such as characteristics of disease and properties of the information and communication. Access to medical information and perceived control over disease management were the top-scoring factors influencing the use of eHealth, indicating a strong need for empowerment. This latter also emerged from our qualitative findings. Correct, complete, and secure data were also of high importance to patients, confirming the results of previous research in dermatology patients [36,37]. Hence, it is important to facilitate patients’ empowerment when implementing eHealth and provide a safe digital infrastructure with complete information. Among the least important scoring factors influencing the use of eHealth were having digital skills, having the right equipment, and receiving support. This is contrary to many other studies reporting these factors as important barriers to the use of eHealth [1,29,36]. Although we did not classify these factors as facilitators or barriers, but instead ranked their relative importance, they were not ranked as important in the use of eHealth. This could be due to our study population being biased, as our study participants might have been more inclined to participate if they were interested in eHealth and already being digitally skilled. Alternatively, it is also possible that patients have become more digitally skilled or more in possession of the right equipment due to the COVID-19 pandemic [38].

### Strengths and Limitations

The use of the Citizen Platform method allowed participants to be more informed about the topic of eHealth and, therefore, were more able to give their opinions and views on the subject. Furthermore, this method allows for the collection of both quantitative and qualitative data, with patients being able to interact with each other, thereby increasing qualitative output. The theory-driven approach in this study by using the COM-B model allowed us to systematically assess factors of influence in the use of eHealth. Furthermore, the model can be used to develop interventions targeting the most important factors. As seen in previous research, the use of citizen science might contribute to more effective implementation [39,40]. However, there are also limitations to be acknowledged for our study. The research design (with 4 parallel focus groups) required the presence of multiple moderators of which some were less experienced. We tried to mitigate the risk of lower quality data due to this in several ways: (1) we standardized the focus group methods by using a topic guide and extensive instruction and discussion before and during the meetings; (2) an experienced moderator was present, who walked around between groups during the parallel focus group sessions, supervising the moderation of groups that needed support; and (3) 2 less experienced moderators were paired together in moderating 1 group. Due to these actions and considering the quality of data we collected, we think that the effect on our findings, if any, is very small. Due to time constraints before and during the assignment for RQ2, there was less room for in-depth exploration, and therefore no quantitative or thematic analysis could be performed. Furthermore, we defined hybrid as a combination of both F2F and digital information or communication. However, during analysis, it was noticed that some participants perceived F2F as conversing with an HCP and digital as mainly reading on a website. Therefore, caution has to be taken when interpreting the quantitative results of preference for communication method, as a preference for F2F may have been overestimated. As the group assignments were performed in focus groups simultaneously, we were unable to iteratively assess data saturation for RQ3a. However, code saturation is expected to occur from 4 focus groups onwards (we had 4 focus groups performing the same assignment), indicating new themes are unlikely to be found [41]. Additional focus groups are advised in future research, as meaning saturation is expected from 4 up to 8 focus groups, thereby fully exploring all insights and nuances [41]. We expect data to be generalizable to other patients with chronic diseases, as we included participants with varying demographics, including a range of age, conditions, disease duration, health literacy, and digital skills. A large proportion of participants were highly educated, however, indicating a possible selection bias, as these patients might already be more digitally skilled or have a higher health literacy. Nonetheless, we put considerable effort into recruiting patients who were less enthusiastic and digitally skilled, who were present in our study population, thereby reducing high selection bias. Furthermore, although we organized the meetings outside of working hours to accommodate as many age groups as possible, we had a relatively high mean age of 67.4 (SD 10.6) years, compared to for example, a median age of onset for rheumatoid arthritis of 45 years in women (50 in men) [42] and a mean age of onset for knee osteoarthritis of 53.5 (SD 14.4) years [43]. This could indicate some additional selection bias. However, this might not have impacted our study results, as the opposite was true for our expectation that digital skills and access to equipment might be important to older patients in the use of eHealth.

### Clinical Implications

The results provide several targets to enhance the use of eHealth in a hospital setting, thereby stimulating and shaping the digital transformation that is needed for sustainable future health care. (1) Individual patients’ needs and preferences should be assessed and reassessed throughout their care pathway, due to the variability of needs and preferences in individual patients, types of information, and moments in the care pathway. (2) eHealth channel use should be tailored to the specificity and impact of information, and a variety of digital channels with a gradient of interaction possibilities should be made available. (3) Requirements such as digital skills and having internet might become less important to focus on in the future, as probably more people own a device and are becoming more digitally skilled. Improving eHealth use by patients may be achieved by providing patients access to correct and safe (medical) information and more control over their care. This final implication may not be generalizable to all settings due to differences in digital access or available staff, reimbursement policies, and other factors influencing the use of eHealth, such as travel distance to a hospital.

### Conclusions

Patients identified opportunities for using eHealth during all stages of their care pathway. Preferences for eHealth channels varied between patients and touchpoints in their care pathway, implicating that multiple channels need to be available. Multiple factors have been identified that influenced the use of eHealth, including the relative importance of factors and providing targets including priorities for eHealth implementation.

## Appendix

Multimedia Appendix 1 Time schedule of the interactive research days.

Multimedia Appendix 2 Topic list for the qualitative assignments.

Multimedia Appendix 3 Thematic analysis approach used for RQ3a.

Multimedia Appendix 4 The homework assignment and explanation of the factors identified from the answers given.

Multimedia Appendix 5 The Q-methodology sorting table that was used for this study.

## Abbreviations

COM-B: Capability, Opportunity, Motivation, and Behavior Model
F2F: face to face
HCP: health care provider
MSD: musculoskeletal disorder
RQ: research question
