# Supplementary material for: Preferences of Patients With Musculoskeletal Disorders Regarding the Timing and Channel of eHealth and Factors Influencing Its Use: Mixed Methods Study
Source: JMIR Hum Factors. 2023 Sep 27;10:e44885. doi: 10.2196/44885 (PMC10568401; doi:10.2196/44885)
Supplement: Multimedia Appendix 1 [file humanfactors_v10i1e44885_app1.doc]

**Multimedia Appendix 1: Time schedule of the interactive research days**

**March 12th, 2022**

10.00 AM Introduction

10.30 AM Lecture: “What healthcare can learn from …..”

10.55 AM COFFEE BREAK

11.10 AM Exercise 1: Identifying touchpoints

12.30 PM LUNCH BREAK

13.15 PM Exercise 2: Digital/physical/hybrid preference for the touchpoints (Research question 1 (quantitative part) and research question 3a (qualitative part))

14.30 PM TEA BREAK

15.00 PM Exercise 3: Preference for communication channel for the touchpoints (Research question 2)

15.30 PM Explanation homework exercise (Exercise 4)

15.40 PM CONCLUSION

**March 19th, 2022**

10.00 AM Introduction

10.15 AM Lecture: “Implementation of Technology & Innovation”

10.50 AM Exercise 5 part 1: Explanation of the factors derived from the homework exercise

11.10 AM COFFEE BREAK

11.30 AM Exercise 5 part 2: Ranking factors: What is most important to you? (Research question 3b)

12.15 PM LUNCH BREAK

13:00 PM Summary of the morning + what can we do about it?

13.15 PM Exercise 6: Time for action! (not used for analysis)

14.15 PM TEA BREAK

14.35 PM Pitching ideas of exercise 6

15:05 PM Evaluation

15.30 PM CONCLUSION
