# Supplementary material for: Preferences of Patients With Musculoskeletal Disorders Regarding the Timing and Channel of eHealth and Factors Influencing Its Use: Mixed Methods Study
Source: JMIR Hum Factors. 2023 Sep 27;10:e44885. doi: 10.2196/44885 (PMC10568401; doi:10.2196/44885)
Supplement: Multimedia Appendix 2 [file humanfactors_v10i1e44885_app2.doc]

**Multimedia Appendix 2: Topic list for the qualitative assignments**

*Assignment 1: Identifying touchpoints, topics to ask about when output is low*

1. First symptoms (attention: this only concerns things that are related to the hospital)

- Need for information about the symptoms, possible diagnosis
- Information about the hospital and treatments
- Appreciation of the hospital and healthcare providers (by other patients)
- Outcomes of the treatments, waiting times

2. First contact and diagnosis:

- Signing up at the hospital (providing information, making a photo)
- Conversation abou the symptoms and diagnosis with the doctor
- Sharing information about recent symptoms
- Providing information from other healthcare providers
- Sharing an overview of current medication with the hospital
- Conversation about coping with the disease
- Referrals to other departments
- Receiving the actual diagnosis

3. Treatment decisions:

- Information regarding treatment options and pros/cons
- Sharing information about personal situation/preferences
- Conversation with doctor about treatment options

4. Treatment

- Filling in questionnaires regarding treatment (e.g. preoperative screening, or about quality of life, functioning.)
- Planning treatment/surgery
- Receiving practical information regarding treatment (where to be, duration, what to expect)
- Information about how to prepare for a surgery (exercises, diet etc)
- De treatment itself (surgery, physical therapy, conversation with HCP(s), infusion therapy)
- Conversation with HCP about (the effect of/progress) of treatment.
- Questions about side effects/complications
- Sharing information about increasing symptoms/effect of the treatment
- Ordering medication
- Pick up medication
- Passing down changing in personal information (new address, changing appointment etc.)
- Questions regarding personal situation, coping with the disease

5. Aftercare:

- Check-up appointment with the doctor (e.g. when disease activity is low, or to check after a surgery)
- Monitoring and sharing information about symptoms/complications

*Assignment 2a: Qualitative part of voting for digital/F2F/hybrid (RQ3a)*

1. Why did you choose for this method? Can you elaborate?
2. Why did you not choose for the other method(s)?
3. Is there a particular reason why it is different for this touchpoint than the other?

*Assignment 3: Digital channel preferences (RQ2)*

1. For this touchpoint, what channel(s) do you prefer? Why this one?
2. Why are other channels not suited for this touchpoint?
3. Are there any channels you can think of, even outside of healthcare, that might be suitable for this touchpoint?
