# Supplementary material for: Preferences of Patients With Musculoskeletal Disorders Regarding the Timing and Channel of eHealth and Factors Influencing Its Use: Mixed Methods Study
Source: JMIR Hum Factors. 2023 Sep 27;10:e44885. doi: 10.2196/44885 (PMC10568401; doi:10.2196/44885)
Supplement: Multimedia Appendix 3 [file humanfactors_v10i1e44885_app3.doc]

**Multimedia Appendix 3: Thematic analysis approach used for RQ3a**

Using the thematic analysis approach by Braun & Clark, researchers (JvdV and LV) (1) familiarized themselves with the transcribed data and (2) identified relevant fragments which were semantically open coded. Open coding was performed independently by two authors (JvdV and LV) and differences were discussed until consensus was reached. From step 3 onwards, a third author (BvdB) was involved in the coding process. (3) Following open coding, axial coding was done in which open codes were gathered to generate potential subthemes on a higher latent level. (4) Initial subthemes were reviewed and refined by checking whether subthemes were distinct and work in relation to the coded text fragments. Subsequently, subthemes were further categorized into a higher latent level to become main themes. (5) Main themes were further defined and renamed by noting down the specific story each theme tells, providing clear definitions per theme. (6) Finally, text fragments supporting the content of the theme were selected, and a descriptive story was written.
