# Supplementary material for: Preferences of Patients With Musculoskeletal Disorders Regarding the Timing and Channel of eHealth and Factors Influencing Its Use: Mixed Methods Study
Source: JMIR Hum Factors. 2023 Sep 27;10:e44885. doi: 10.2196/44885 (PMC10568401; doi:10.2196/44885)
Supplement: Multimedia Appendix 4 [file humanfactors_v10i1e44885_app4.doc]

**Multimedia Appendix 4: The homework assignment and explanation of the factors identified from the answers given**

**Reasons to (not) use eHealth**

We are interested in topics that stimulate or hinder your use of eHealth. To help you think about this, we have made an example about things that stimulate or hinder people in eating healthy.

EXAMPLE: Eating healthy

Food influences people’s health. A lot of people strive to eat healthy.

In doing so, there are things that stimulate eating healthy. Think about: knowing what healthy food is. Having time to prepare a healthy meal. A partner that likes to eat healthy. Being convinced that eating health makes you feel better. Feeling fitter by eating healthy.

Next to that, there are things that hinder people to eat healthy. Think about: Not knowing what healthy food is. A partner eating unhealthy food. Being used to a certain eating pattern. Cost of eating healthy. Liking the taste of unhealthy food. Having difficulty preparing a healthy meal.

After this example, we are interested in things that stimulate or hinder your use of eHealth of the hospital. Write down the first thing that comes to mind, there are no wrong answers. Write down:

1. 3 or more things that stimulate your use of eHealth
2. 3 or more things that hinder your use of eHealth.
