# Supplementary material for: Preferences of Patients With Musculoskeletal Disorders Regarding the Timing and Channel of eHealth and Factors Influencing Its Use: Mixed Methods Study
Source: JMIR Hum Factors. 2023 Sep 27;10:e44885. doi: 10.2196/44885 (PMC10568401; doi:10.2196/44885)
Supplement: Multimedia Appendix 5 [file humanfactors_v10i1e44885_app5.doc]

**Multimedia Appendix 5: The Q-methodology sorting table that was used for this study**

**Figure S1. Q-methodology sorting table for 23 factors.** The normally distributed Q-sorting table that was used during this study. Participants sorted the cards in the squares, with the middle being neutral. Sorting factors on the right or left side meant that these factors were important and least important for use of eHealth respectively.
